# An inter-laboratory study of DNA-based identity, parentage and species testing in animal forensic genetics

**DOI:** 10.1080/20961790.2021.1886679

**Published:** 2021-07-19

**Authors:** Sreetharan Kanthaswamy, Torsten Brendel, Luis Cancela, Denise A. Andrade de Oliveira, Bertram Brenig, Carmen Cons, Julian A. Crespi, Markéta Dajbychová, Andreas Feldl, Tomohito Itoh, Vincenzo Landi, Amparo Martinez, Malgorzata Natonek-Wisniewska, Robert F. Oldt, Anna Radko, Oscar Ramírez, Clementina Rodellar, Manuel Ruiz-Girón, David Schikorski, María Elena Turba, Guillermo Giovambatista

**Affiliations:** a School of Mathematics and Natural Sciences, Arizona State University (ASU) at the West Campus, Glendale, AZ, USA; b California National Primate Research Center, University of California, Davis, CA, USA; c Evolutionary Biology Graduate Program, School of Life Sciences, Arizona State University, Tempe, AZ, USA; d Eurofins Genomics Europe Applied Genomics GmbH, Ebersberg, Germany; e IDENTITAS, Montevideo, Uruguay; f Laboratório de Genética, Escola de Veterinária da UFMG, Belo Horizonte, Brazil; g Institute of Veterinary Medicine, University of Goettingen, Goettingen, Germany; h Facultad de Veterinaria, Laboratorio de Genética Bioquímica (LAGENBIO), Instituto Agroalimentario de Aragón-IA2 (Universidad de Zaragoza-CITA), Zaragoza, Spain; i Facultad de Ciencias Veterinarias UNLP, IGEVET – Instituto de Genética Veterinaria (UNLP-CONICET LA PLATA), La Plata, Argentina; j Genomia s.r.o., Plzeň, Czech Republic; k Maebashi Institute of Animal Science, Livestock Improvement Association of Japan (LIAJ), Maebashi, Japan; l Department of Veterinary Medicine, Animal Breeding and Genetics, University of Bari “Aldo Moro”, Valenzano, Italy; m Department of Genetics, Faculty of Veterinary Sciences, University of Córdoba, Córdoba, Spain; n Department of Animal Molecular Biology, National Research Institute of Animal Production, Balice, Poland; o Vetgenomics SL, Barcelona, Spain; p Laboratorio de Biología Molecular y Genómica, Hispalis Biolab S.L.U., Seville, Spain; q Laboratoire LABOFARM-GENINDEXE, LOUDEAC Cedex, France; r Genefast SRL, Forlì, Italy

**Keywords:** Forensic sciences, forensic genetics, comparison test, forensic DNA analysis, individual identification, parentage test, species test

## Abstract

The probative value of animal forensic genetic evidence relies on laboratory accuracy and reliability. Inter-laboratory comparisons allow laboratories to evaluate their performance on specific tests and analyses and to continue to monitor their output. The International Society for Animal Genetics (ISAG) administered animal forensic comparison tests (AFCTs) in 2016 and 2018 to assess the limitations and capabilities of laboratories offering forensic identification, parentage and species determination services. The AFCTs revealed that analyses of low DNA template concentrations (≤300 pg/µL) constitute a significant challenge that has prevented many laboratories from reporting correct identification and parentage results. Moreover, a lack of familiarity with species testing protocols, interpretation guidelines and representative databases prevented over a quarter of the participating laboratories from submitting correct species determination results. Several laboratories showed improvement in their genotyping accuracy over time. However, the use of forensically validated standards, such as a standard forensic short tandem repeat (STR) kit, preferably with an allelic ladder, and stricter guidelines for STR typing, may have prevented some common issues from occurring, such as genotyping inaccuracies, missing data, elevated stutter products and loading errors. The AFCTs underscore the importance of conducting routine forensic comparison tests to allow laboratories to compare results from each other. Laboratories should keep improving their scientific and technical capabilities and continuously evaluate their personnel’s proficiency in critical techniques such as low copy number (LCN) analysis and species testing. Although this is the first time that the ISAG has conducted comparison tests for forensic testing, findings from these AFCTs may serve as the foundation for continuous improvements of the overall quality of animal forensic genetic testing.

## Key points

Comparison tests allow laboratories to evaluate their analyses for accuracy and reliability.

Two forensic identification, parentage and species determination comparison tests were performed.

The study showed that the LCN DNA analysis represented a significant challenge to most laboratories.

Lacking familiarity with species tests curbed most laboratories from reporting accurately.

A reliance on forensically validated testing standards may have prevented some of the common errors.

## Introduction

Forensic genetic testing of animal biomaterials is a firmly established investigative approach. However, laboratories providing these services must continue to take rigorous quality assurance measures to gene­rate reliable and accurate results. The International Society for Animal Genetics (ISAG) implemented two animal forensic comparison tests (AFCTs) in 2016 and 2018 to methodologically and robustly evaluate each participating laboratory’s ability to: (1) genotype dog short tandem repeat (STR) markers for identification and parentage resolution and (2) determine the species of test samples. Conducted under the quality assurance standards for DNA analysis recommended by the DNA Advisory Board [1] and recom­mendations made for animal DNA forensic testing [2], the AFCTs mark the first instance that the ISAG has conducted comparison tests based on forensic testing standards among its member laboratories.

Although standardized genetic tests are critical for sharing information and combining datasets, there are no ISAG-recommended markers or forensic testing protocols [2]. Consequently, each partici­pating laboratory was permitted to use domestic dog genotyping panels of their choice to generate individual identification and parentage outcomes. Similarly, the laboratories were also allowed to choose their preferred method of species identification. Thus, this study aimed to learn if different standard operating protocols used by the participa­ting laboratories would limit their ability to produce acceptable results and affect their conclusions when analyzing the same sample. Supplementary File 1 shows the AFCT survey questions provided to the laboratories in 2016 and 2018.

## Materials and methods

### 2016 AFCT

Table 1 lists the volume and concentration for each DNA test sample, solubilized in Tris–EDTA buffer, used for the AFCTs. The 2016 identification test included one diluted (150 pg/µL) and two undiluted (25 ng/µL) aliquots of domestic dog (*Canis lupus familiaris*) DNA samples that were isolated from the same individual dog. The species test in 2016 involved one undiluted (50 ng/µL) cattle (*Bos taurus*) DNA sample. All four test samples were shipped to 25 labo­ratories that applied to participate in the 2016 AFCT.

**Table 1.** Details on DNA test samples included in the 2016 and 2018 animal forensic comparison tests (AFCTs).

AFCT	Sample	Species	Purpose^a^	Quality	Concentration (volume)
2016	1	*Canis lupus familiaris*	M	Undiluted	25 ng/µL (50 µL)
	2	*Canis lupus familiaris*	M	Diluted	150 pg/µL (50 µL)
	3	*Canis lupus familiaris*	M	Undiluted	25 ng/µL (50 µL)
	4	*Bos taurus*	S	Undiluted	50 ng/µL (50 µL)
2018	1	*Canis lupus familiaris*	M	Diluted	300 pg/µL (30 µL)
	2	*Canis lupus familiaris*	M	Undiluted	50 ng/µL (30 µL)
	3	*Canis lupus familiaris*	M	Undiluted	50 ng/µL (30 µL)
	4	*Canis lupus familiaris*	P	Undiluted	10–20 ng/µL (30 µL)
	5	*Canis lupus familiaris*	P	Undiluted	10–20 ng/µL (30 µL)
	6	*Canis lupus familiaris*	P	Undiluted	10–20 ng/µL (30 µL)
	7	*Bos taurus*	S	Undiluted	50 ng/µL (30 µL)
	8	*Gadus morhua*	S	Undiluted	50 ng/µL (30 µL)

^a^M and P indicate dog DNA samples designated for forensic identification and parentage tests, respectively; S indicates DNA from an unknown species for species determination.

### 2018 AFCT

The 2018 AFCT involved an identification test, a parentage test and a species test (Table 1). As with the 2016 AFCT, the 2018 identification test comprised one diluted (300 pg/µL) and two undiluted (50 ng/µL) DNA samples from the same animal. The 2018 AFCT also implemented a parentage test involving undiluted DNA sample concentrations ranging from 10 ng/µL to 20 ng/µL from a parent–offspring trio. The 2018 AFCT also included two separate species tests comprising undiluted cattle (50 ng/µL) and fish (Atlantic cod; *Gadus morhua*) (50 ng/µL) DNA. For the 2018 AFCT, a set of eight DNA samples, including the two species test samples, were shipped to 26 laboratories. Ten of these laboratories had also participated in the 2016 AFCT.

The 2018 cattle and fish species test samples reflect the range of taxa often encountered during animal forensic investigations. Indeed, the 2018 survey confirmed that the majority of laboratories routinely analyze a vast variety of samples from different species: domestic dogs (100%), various bird species including chickens and racing pigeons (100%), horses and donkeys (95%), cattle (87.7%), sheep (87.7%), various fish species (84.6%), goats (64.3%), domestic cats (57.1%), various wild mammalian species (53.9%), swine (42.1%), insects (7.7%), protozoa (7.7%) and reptiles (0.09%).

### Sample preparation, shipment and analysis

DNA samples used for the 2016 and 2018 AFCTs were extracted from dog whole blood, as well as cattle and fish meat, using the Chemagic™ MSM I System (Perkin Elmer, Waltham, MA, USA) at the Duty Laboratory (Eurofins Genomics, Ebersberg, Germany). DNA concentrations were determined using a DropSense 96 polychromatic spectrophotometer (Trinean, Pleasanton, CA, USA), and the DNA sample dilutions were performed with a Hamilton Microlab Star Plus Liquid Handling System (Hamilton, Reno, NV, USA). The laboratories participating in the 2016 and 2018 AFCTs were located in Argentina, Czech Republic, France, Germany, Italy, Japan, the Netherlands, Poland, South Africa, Slovenia, Spain, USA and Uruguay. Ten laboratories submitted their results in 2016 and 2018 tests. All AFCT results and survey data were submitted by these laboratories directly to the Computer Laboratory (Identitas Laboratory, Montevideo, Uruguay) for analysis.

## Results and discussion

Eighteen (72%) of the 25 laboratories that received the test samples for the 2016 AFCT reported their results, whereas in 2018, 19 (73%) of the 26 laboratories that received the test samples reported their results. Over 55.17% (16/29) of all of these laboratories (*N* = 29) belonged to university or government institutions, while approximately 44.83% (13/29) were private laboratories. The services provided by these laboratories include parentage which represents the vast majority of caseloads (93.33%, 14/15), animal theft (80.00%, 12/15), contamination/adulteration (66.67%, 10/15), illegal traffic/poaching (53.33%, 8/15), animal attack (46.67%, 7/15), animal cruelty (46.67%, 7/15), human forensic investigation (40.00%, 6/15), traffic accidents (40.00%, 6/15) and horse doping (66.67%, 10/15). The surveys showed that the clienteles of the participating laboratories included animal breeders (75.00%, 9/12), public administration units (75.00%, 9/12), law enforcement units (66.67%, 8/12), food and beverage industries (50.00%, 6/12), animal breeding associations (33.33%, 4/12), other private laboratories (25.00%, 3/12), forensic practitioners (16.67%, 2/12), insurance companies (16.67%, 2/12), trading companies (16.67%, 2/12), the aviation industries (8.33%, 1/12), hunting societies (8.33%, 1/12) and various other companies (16.67%, 2/12).

In 2016, 15 laboratories produced dog STR geno­types using the ISAG core parentage panel (http://www.isag.us/Docs/consignmentforms/2005ISAGPanelDOG.pdf), and three laboratories used the Canine Genotypes™ Panel 1.1 Kit (Thermo Fisher Scientific, Waltham, MA, USA). Since only one laboratory relied on the Canine Genotypes™ Panel 2.1 Kit (Thermo Fisher Scientific) and one other laboratory used the Can-ID™ (Vetgenomics, Barcelona, Spain) SNP panel in 2018, the results from these two labo­ratories were not used in this study. Table 2 displays the STRs included in Panel 1.1 and the core ISAG panel. In 2018, 10 laboratories used the ISAG core STRs, eight used Panel 1.1, and one used both panels. The ISAG core markers (with 21 STRs and an amelogenin gene sex-typing marker) and the Canine Genotypes™ Panel 1.1 (with 18 STRs and an amelogenin gene sex-typing marker) were developed for laboratories that provide dog parentage and identity testing services. The 18 STRs in Panel 1.1 are among the 21 ISAG core markers. FH2054 is the only tetranucleotide repeat STR in Panel 1.1. All other STRs in this kit and the ISAG core panel contain dinucleotide repeat motifs [3].

**Table 2.** The percentage of relative genotyping accuracy values for each STR in Panel 1.1 and the core ISAG panel were estimated as the ratio between the concordant genotypes and reported genotypes for the 2016 and 2018 AFCTs, respectively.

	Relative genotyping accuracy (%)	
Marker name	2016 AFCT	2018 AFCT
AHT121*	93.83	94.51
AHT137*	70.80	96.70
AHTh130	100.00	92.54
AHTh171*	83.30	91.21
AHTh260*	88.20	91.21
AHTk211*	83.30	92.31
AHTk253*	94.10	100.00
CXX0279*	88.90	91.21
FH2054*	88.90	89.25
FH2848*	94.10	85.71
INRA021*	72.20	91.21
INU005*	72.20	88.37
INU030*	83.30	91.21
INU055*	83.30	91.21
REN105L03	70.00	89.47
REN162C04*	88.90	95.60
REN169D01*	88.20	95.35
REN169O18*	88.90	95.60
REN247M23*	83.30	91.21
REN54P11*	77.80	94.51
REN64E19	100.00	93.42

*The 18 STRs in Panel 1.1 that are part of the core ISAG panel.

Laboratories that reported STR data from the undiluted dog DNA were able to amplify between 19 (eight laboratories) and 23 markers (one laboratory) in 2016, and 19 (three laboratories) to 40 markers (one laboratory) in 2018 (Supplementary File 2). Analyses of undiluted dog DNA samples permitted all but one laboratory to correctly respond to the individual identification test questions (over 97.30%) because of the high percentage of correct STR allele calls. When results from that one laboratory were ignored, both Panel 1.1 and the ISAG core panel performed comparably on undiluted samples by yielding genotyping results that could be readily interpreted. Approximately 84.21% of the parentage test questions which were based on undiluted DNA samples, including paternity and maternity assignments, were correct, mostly because these laboratories considered the DNA-based sex information in their parentage analyses. Correctly assigning the sex proved essential for confirming paternity/maternity and the identification of each animal involved in the parentage tests.

Because the laboratories employed different STR panels, consensus allele sizes and genotypes were not compared across laboratories. However, the relative genotyping accuracy across STRs ranged from 70% to 100% with an average of 85.41% in 2016. In 2018, this value ranged from 85.71% to 100% with an ave­rage of 92.47% (Table 2). Despite the increase in genotyping accuracy from 2016 to 2018, the performance variability across laboratories may be attributed to the lack of an effective allelic ladder, which has yet to be developed and validated for the STR panels used in this study. As allelic ladders present the common alleles of an STR, the size of the alleles of unknown samples can be designated by comparing them to the rungs of the allelic ladder to obtain the most accurate allele assignments possible [2].

Supplementary File 3 presents absolute (Aga) and relative (Rga) STR genotyping accuracy estimates generated from both the 2016 and 2018 AFCTs and also ranks the performance of participating laboratories from the lowest to the highest for each comparison test based on the ISAG’s Genotype Rating System (https://www.isag.us/docs/Rules_CT.pdf?v2). The average Aga and Rga values were 78.08 and 84.59 in 2016 and 85.93 and 90.89 in 2018, respectively. Among the 10 laboratories that participated in both 2016 and 2018, four experienced no change in their high genetic testing performance, while the remaining six showed improvement in their performance with average estimates of Aga and Rga increasing from 85.54 to 96.64 and from 93.10 to 99.82, respectively. Based on their Aga and Rga values, private laboratories performed slightly better than government/academic laboratories in 2016 (Table 3). In 2018, however, the Rga% government/academic laboratories performing was better than their private counterparts. Based on the data presented in Table 3, the estimates of standard deviation within each group were higher than those between groups suggesting that there were high levels of performance variability across government/academic laboratories and academic laboratories, respectively.

**Table 3.** Comparison of average absolute (Aga) and relative genotyping accuracy (Rga) estimates among laboratories (%, mean±SD).

Subject	2016 AFST			2018 AFST		
	*n*	Rga%	Aga%	*n*	Rga%	Aga%
Government/academy	8	77.25±31.79	66.36±27.36	11	91.78±26.06	85.70±28.31
Private	10	97.87±2.66	92.97±6.62	8	89.67±17.71	89.67±20.34

Absolute (Aga) and relative (Rga) values for the short tandem repeats (STRs) included in the International Society for Animal Genetics (ISAG) core panel as well as the Canine Genotypes™ Panel Kit 1.1 (Thermo Fisher Scientific) using ISAG’s formula for STR typing comparison tests (https://www.isag.us/docs/Rules_CT.pdf?v2).

Aga% = (total number of expected genotypes − genotyping errors including no genotype reported)/total number of genotypes.

Rga% = (the number of genotypes reported − total number of genotype errors excluding blanks)/the number of genotypes reported.

The type and rate of genotyping errors that were used to estimate the Aga and Rga values for the 2016 and 2018 AFCTs are presented in Table 4. For both AFCTs, “missing data” due to results being omitted by the laboratories were the most common errors and accounted for 46.00% of all errors detected. For clarity, “missing data” errors were grouped into three categories: STR blanks (no amplification or less efficient amplification of one or more STRs occurred), sample blanks (one or more samples failed to amplify) and genotype blanks (one or more markers failed to amplify during the multiplex reaction). False homozygotes mainly due to stochastic allele dropout arising from diluted DNA samples, incorrect genotype calls due to improper binning of alleles, mistaken alleles, or misidentification of allelic microvariants and false heterozygotes due to stutter products collectively represented 31.23% of the total errors observed. The incidence of elevated stutter peaks from dinucleotide STRs in Panel 1.1 and the ISAG core panel accounted for 5.50% of the total number of errors in both AFCTs. Additionally, typing and nomenclature errors represented approximately 22.77% of the total number of errors observed in this study (Table 4).

**Table 4.** Percentage of different types of genotyping errors in each animal forensic comparison test (AFCT)*.

AFCT	Missing data (%)			Loading error (%)		False homozygotes (%)	False heterozygotes (%)	Incorrect genotypes (%)
	STR blanks	Sample blanks	Genotype blanks	Typing errors	Nomenclature errors			
2016	33.33	0.00	4.76	2.38	15.48	5.95	5.95	32.14
2018	20.48	15.02	15.02	19.45	6.14	2.73	0.68	20.48
Total	25.16	9.54	11.30	13.23	9.54	3.90	2.60	24.73

*Percentages might not total 100% due to rounding.

Forensic genetic analysis regularly depends on trace evidence. Thus, the ability to resolve identity and parentage using low template DNA is particularly critical. The 2016 and 2018 AFCTs, however, exposed the challenges in testing low template DNA samples with concentrations of ≤300 pg/µL. Yet, this concentration is higher than the low copy number (LCN) limit in human forensic DNA analysis, which is 100 pg/µL [4,5]. Most laboratories agreed that the diluted nuclear DNA concentration yielded STR peaks below their ideal detection threshold. Only one labo­ratory successfully reported genotypes with no errors across all 19 STRs that were employed. The remaining laboratories either submitted incorrect STR allele calls (for 1 to 13 markers; Supplementary File 4) or failed to generate any STR amplicons (0% concordance). Several laboratories reported a high prevalence of homozygosity across markers with greater allele size ranges. Unfamiliarity with sensitivity-enhancing techniques [6–8] may have stopped some laboratories from achieving successful LCN DNA analysis. Neither Panel 1.1 nor the ISAG core panel has been validated for forensic DNA testing, including LCN analysis. Because this was the first time many laboratories used Panel 1.1, their lack of famili­arity with this panel also compounded problems with allele assignments and genotype calls. Stutter peaks, mainly those from the dinucleotide STRs in Panel 1.1 and the ISAG core panel, also confounded the genotyping of diluted DNA samples.

Although each participating laboratory had access to the forensically validated Canine Genotypes™ Panel 2.1 Kit, which was developed for standardized forensic DNA typing of domestic dog samples [9–12], only one laboratory opted to use it in 2018. Except for VWF.X, a mostly hexameric marker, and FH3377, a mostly pentameric marker, all other STRs in Panel 2.1 are tetrameric [3]. Therefore, all Panel 2.1 STRs exhibit reduced stutter product formation, which bene­fits sample mixture interpretation and LCN analysis [2,10]. This panel was put through a series of validation steps to further determine its robustness and reliability in forensic DNA typing [3,10,11]. These validation measures included sensitivity, sizing precision, reproducibility, allele dropout, polymerase chain reaction (PCR) artefact characterization (e.g. dye blobs, stutters, split peaks), intra- and inter-locus colour balance, annealing temperature and cycle number studies, peak height ratio determination, mixture analysis (deconvoluting samples from more than one donor), species speci­ficity, concordance, forensic case type sample (e.g. limited and degraded samples) and population stu­dies [3,10,11]. If any of these rigorous development procedures and validation studies were not performed for Panel 1.1 and the core ISAG panel, these marker panels may have been prevented from meeting the quality standards expected for uniform forensic testing protocols.

Biomaterials from a wide variety of species can be encountered during forensic investigations. Because most animal genetic markers are species specific, species confirmation is typically done before genotyping analysis [13,14]. Furthermore, species determination based on genetic testing tends to be more accurate than documentary, physical or biological evidence for identifying, authenticating or tracing the source of biological products, including food and other artefacts [15]. The 2016 species test included one cattle sample, but the 2018 species test included two test samples: cattle and fish. Cumulatively, the 2016 and 2018 species tests involved 56 genetic analyses (three samples per laboratory). Fifteen of the 18 (83%) participating laboratories identified the cattle sample correctly in the 2016 AFCT versus 16 of the 19 (84%) laboratories that participated in the 2018 AFCT. Unfortunately, the remaining laboratories could not resolve the species test successfully.

Over 89% (17/19) of the laboratories answered the species test questions correctly. Several laboratories failed the species test because they had either eliminated cattle as the correct species or experienced potential cross-reactivity with other species, including bovine–equine mixes and bovine–canine–ovine mixes. In contrast, others initially failed the species test because of low DNA quantity and subsequently needed more DNA to conclude the test correctly or did not submit any result. The laboratories employed a variety of species testing methods, including approaches that were better suited for a wide range of target species, such as sequenced-based typing (PCR-SBT) of mitochondrial 16S ribosomal ribonucleic acid (rRNA), cytochrome b (Cytb) or cytochrome c oxidase I (COI) sequences, and others with a narrow range of target species such as allele-specific PCR and species identification by insertion/deletion (SPInDel) assays [16,17]. The limi­ted range of species detection capability is likely why several laboratories could not correctly exclude the different possible species listed in the tests. The 2018 fish species test resulted in 10 laboratories (almost 53%, 10/19) correctly identifying the sample as *Gadus morhua*. The other laboratories did not report any results because either testing for fish species was outside of their scope of expertise or they lacked access to technology such as DNA sequencing that could have enabled them to identify the fish species. Therefore, the species testing evalu­ation of the AFCTs indicates that a lack of famili­arity with species testing protocols, interpretation guidelines and representative databases or the taxa have restricted several laboratories from submitting correct responses for this evaluation [2,18].

## Conclusion

This report describes the results of the 2016 and 2018 AFCTs that were administered for the first time by the ISAG to determine the limitations and capabilities of animal genetic laboratories that provide forensic services worldwide. Data from laboratories that participated in the 2016 and 2018 AFCTs confirmed that six out of 10 of these laboratories’ performances improved with time. Therefore, AFCTs can be used periodically to demonstrate the quality performance of an animal forensic genetic laboratory, as well as serve as a mechanism for critical self-evaluation. The results of the AFCTs are also an impetus for the ISAG Animal Forensic Genetics Standing Committee to vigorously and collaboratively develop and validate uniform forensic testing protocols, such as a standard forensic STR kit, prefe­rably with an allelic ladder, and stricter guidelines for STR analysis. These initiatives could help curtail some of the common, but avoidable, issues observed in the AFCTs, such as incorrect genotyping, missing data, and loading errors. Laboratories providing animal forensic genetic testing services should keep improving their scientific and technical capabilities and continuously evaluate their personnel’s know­ledge, skills and abilities to enhance their competency with important animal forensic techniques and technologies, including LCN analysis and species testing. The use of forensically validated approaches will facilitate uniform techniques and promote data sharing so that laboratories worldwide can develop their skills and abilities and provide quality animal forensic genetics services.

## References

[1] DNA Advisory Board (DAB). Quality assurance standards for forensic DNA testing laboratories. Forensic Sci Commun. 2000;2.

[2] Budowle B, Garofano P, Hellman A, et al. Recommendations for animal DNA forensic and identity testing. Int J Legal Med. 2005;119:295–302. 15834735 10.1007/s00414-005-0545-9

[3] Kanthaswamy S, Oldt RF, Montes M, et al. Comparing two commercial domestic dog (*Canis familiaris*) STR genotyping kits for forensic identity calculations in a mixed-breed dog population sample. Anim Genet. 2019;50:105–111. 30592310 10.1111/age.12758

[4] Gill P, Whitaker J, Flaxman C, et al. An investigation of the rigor of interpretation rules for STRs derived from less than 100 pg of DNA. Forens Sci Int. 2000;112:17–40. 10.1016/s0379-0738(00)00158-4 10882828

[5] Gill P. Application of low copy number DNA profiling. Croat Med J. 2001;42:229–232. 11387628

[6] Balding DJ, Buckleton J. Interpreting low template DNA profiles. Forens Sci Int Genet. 2009;4:1–10. 10.1016/j.fsigen.2009.03.003 19948328

[7] Budowle B, Eisenberg AJ, van Daal A. Validity of low copy number typing and applications to forensic science. Croat Med J. 2009;50:207–217. 19480017 10.3325/cmj.2009.50.207 PMC2702736

[8] Gill P, Puch-Solis R, Curran J. The low-template-DNA (stochastic) threshold—its determination relative to risk analysis for national DNA databases. Forensic Sci Int Genet. 2009;3:104–111. 19215879 10.1016/j.fsigen.2008.11.009

[9] Kanthaswamy S, Tom BK, Mattila AM, et al. Canine population data generated from a multiplex STR kit for use in forensic casework. J Forens Sci. 2009;54:829–840. 10.1111/j.1556-4029.2009.01080.x 19486242

[10] Dayton M, Koskinen MT, Tom BK, et al. Developmental validation of short tandem repeat reagent kit for forensic DNA profiling of canine bio­logical material. Croat Med J. 2009;50:268–285. 19480022 10.3325/cmj.2009.50.268 PMC2702741

[11] Tom BK, Koskinen MT, Dayton M, et al. Development of a nomenclature system for a canine STR multiplex reagent kit. J Forens Sci. 2010;55:597–604. 10.1111/j.1556-4029.2010.01361.x 20345775

[12] Ogden R, Mellanby RJ, Clements D, et al. Genetic data from 15 STR loci for forensic individual identification and parentage analyses in UK domestic dogs (*Canis lupus familiaris*). Forens Sci Int Genet. 2012;6:e63–e65. 10.1016/j.fsigen.2011.04.015 21600864

[13] Linacre A, Tobe SS. An overview to the investigative approach to species testing in wildlife forensic science. Investig Genet. 2011;2:1–9. 10.1186/2041-2223-2-2 PMC3032691 21232099

[14] Kanthaswamy S, Premasuthan A. Quadriplex real-time PCR (qPCR) assay for human-canine-feline species identification and nuclear DNA quantification. Forens Sci Int Genet. 2012;6:e97–e98. 10.1016/j.fsigen.2011.09.001 21962833

[15] Kanthaswamy S. Review: domestic animal forensic genetics—biological evidence, genetic markers, analytical approaches and challenges. Anim Genet. 2015;46:473–484. 26364867 10.1111/age.12335

[16] Pereira F, Carneiro J, Matthiesen R, et al. Identification of species by multiplex analysis of variable–length sequences. Nucleic Acids Res. 2010;38:e203. 20923781 10.1093/nar/gkq865 PMC3001097

[17] Carneiro J, Pereira F, Amorim A. SPInDel: a multifunctional workbench for species identification using insertion/deletion variants. Mol Ecol Resour. 2012;12:1190–1195. 22978681 10.1111/1755-0998.12011

[18] Linacre A, Gusmão L, Hecht W, et al. ISFG: recommendations regarding the use of non-human (animal) DNA in forensic genetic investigations. Forens Sci Int Genet. 2011;5:501–505. 10.1016/j.fsigen.2010.10.017 21106449

